# Severe Thrombocytopenia as a Manifestation of COVID-19 Infection

**DOI:** 10.3390/jcm11041088

**Published:** 2022-02-18

**Authors:** Mihaela Mocan, Roxana Mihaela Chiorescu, Andrada Tirnovan, Botond Sandor Buksa, Anca Daniela Farcaș

**Affiliations:** 1 Internal Medicine Department, Iuliu Hatieganu University of Medicine and Pharmacy, 400012 Cluj-Napoca, Romania; mihaela.mocan@gmail.com (M.M.); ancafarcas@yahoo.com (A.D.F.); 2 Department of Internal Medicine, Emergency Clinical County Hospital, 400006 Cluj-Napoca, Romania; andrada.tirnovan@gmail.com (A.T.); buksaboti@yahoo.com (B.S.B.); 3 Department of Cardiology, Emergency Clinical County Hospital, 400006 Cluj-Napoca, Romania

**Keywords:** severe thrombocytopenia, COVID-19 infection, disseminated intravascular coagulation

## Abstract

Clinical manifestations of COVID-19 infection can range from an asymptomatic clinical form to acute respiratory distress depending on the virus gateway, viral load, host immunity, and existing comorbidities. Some patients with COVID-19 infection can present hematological changes depending on the patient’s immune response and the severity of the infection. We present two different manifestations of thrombotic disorders related to COVID-19: one severe form of immune thrombocytopenia in a young woman with no comorbidities and a severe form of thrombocytopenia along with disseminated intravascular coagulation and acute urinary obstructive disease. Interestingly, both patients presented no signs of COVID-19 pneumonia. Failure to diagnose thrombocytopenia rapidly may lead to severe complications. Management with immunosuppressive corticosteroids in high doses should carefully balance the risk of bleeding versus deterioration due to infection.

## 1. Introduction

Clinical manifestations of COVID-19 infection can range from an asymptomatic clinical form to acute respiratory distress depending on the virus gateway, viral load, host immunity, and existing comorbidities [1]. Clinical respiratory and gastrointestinal manifestations are the most common in COVID-19-positive patients, but extrapulmonary manifestations such as neurological, dermatological, and cardiovascular have also been reported, usually associated with respiratory manifestations and only rarely present independently of the respiratory infection [2].

COVID-19 is also responsible for a spectrum of manifestations that include the hematopoietic system and have life-threatening consequences [3]. It is a severe inflammatory disease that can trigger thrombus formation and pulmonary embolism or in situ pulmonary thrombosis [4]. Moreover, patients affected by the pandemic can develop platelet count changes such as thrombocytopenia. Thrombocytopenia can be detected in almost half of the patients infected by SARS-CoV-2 and in almost 95% of those critically ill [5]. As far as we know, a severe drop in platelet count is only rarely reported and only in association with immune thrombocytopenic purpura [6].

Given the rare incidence of such association, we share our clinical experience regarding COVID-19-induced thrombocytopenia and thrombocytopenic purpura, highlighting the therapeutic approach and the patients’ clinical evolution.

## 2. Clinical Experience

### 2.1. Case Report 1

**Figure 1:**
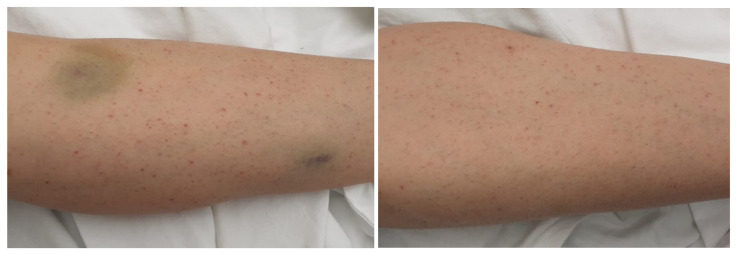
Ecchymosis and skin purpuric lesions of the lower limbs.

We present the case of a 35-year-old female patient who presented in the Emergency Department (ER) for gingivorrhagia, generalized skin purpura, and lower limb bruising. The symptoms had suddenly started 4 days before her presentation to the ER. The family medical history was unimportant, but she had a history of bilateral breast implant performed 5 years ago. The physical examination revealed good general condition, normal weight, petechiae, and diffuse bruises mainly on the lower limbs (Figure 1). The examination of the respiratory and cardiovascular systems did not reveal any pathological changes, SaO_2_ in the atmospheric air being 100%, BP = 115/64 mm Hg, AV = 85 b/min.

Laboratory tests performed in ER showed severe thrombocytopenia (platelets = 1000/mm^3^), and the antigen rapid test for SARS-CoV-2 from the nasal swab was positive at admission. 

The clinical diagnosis was severe thrombocytopenia and thrombocytopenic purpura.

The differential diagnosis was made by considering other conditions, such as:

- False thrombocytopenia;
- Common causes of thrombocytopenia: pregnancy, drug-induced thrombocytopenia (heparin, antibiotics, quinidine, etc.), viral infections (HIV, infectious mononucleosis, hepatitis B, C), and hypersplenism;
- Other causes of thrombocytopenia, which are similar to the immune thrombocytopenic purpura (myelodysplasia, congenital thrombocytopenia, thrombotic thrombocytopenic purpura, hemolytic–uremic syndrome, or DIC);
- Thrombocytopenia associated with other conditions, such as autoimmune conditions (e.g., systemic lupus erythematosus) and lymphoproliferative disorders (chronic lymphocytic leukemia, non-Hodgkin’s lymphoma, etc.).

Laboratory investigation during the hospitalization showed bicytopenia: mild leukopenia, and severe thrombocytopenia (1000/mm^3^). The D-dimers and the fibrinogen were within normal parameters, excluding consumption related to DIC. The inflammatory markers, namely C-reactive protein and ferritin, were found non-reactive. The pregnancy test was negative. The markers for influenza, syphilis, hepatitis B, hepatitis C, and HIV were also negative. The SARS-CoV-2 RT-PCR test confirmed the COVID-19 infection. The anti-nuclear antibodies, anti-smooth muscle antibodies, anti-platelet antibodies, and circulating immune complexes were also found to be nonreactive. The abdominal ultrasound did not show any hepatic or splenic morphological changes. Due to the presence of COVID-19 infection, a lung radiograph was performed, which did not show any pathological changes.

A hematological examination was performed, which helped establish the diagnosis of immune thrombocytopenic purpura caused by SARS-CoV-2 infection. Bone marrow puncture was not indicated because of the patient’s young age and the non-existent comorbidities. However, performing a bone marrow puncture might be considered in the future if the patient presents low sensitivity to the first line treatment (glucocorticoids), if splenectomy is required for treatment, or if a relapse of the disease occurs after complete remission.

The final diagnosis was a mild form of COVID-19, thrombocytopenic purpura in the context of COVID-19, and mild thrombocytopenia.

The goal of treatment during hospitalization was to restore platelet mass.

Drug treatment during hospitalization consisted of administration of corticosteroids (dexamethasone 16 mg/day), vitamins B and C, and gastric protectants. Development during hospitalization slowly turned favorable (the increase in platelets was observed after one week of treatment and their serum value normalized after two weeks) with the improvement of purpura in the lower limbs along with the gradual increase in platelet count.

The patient was discharged after 14 days, and the recommendations on discharge consisted of a low sodium diet and continuation of treatment with Medrol for another 10 days (48 mg/day), followed by a gradual decrease in doses and gastric protectants. The patient was under hematological observation and her development was favorable. At 3 and 6 months after discharge, hematological evaluation revealed no pathological modification in the blood smear.

The particularities of the case are:

- Severe immune thrombocytopenic purpura was an isolated manifestation associated with a mild form of SARS-CoV-2 infection;
- Bleeding/hemorrhage was absent.

### 2.2. Case Report 2

A male patient, 86 years old, with no significant personal pathology, presented in ER for sudden appearance of purpuric lesions on lowers limbs, macroscopic hematuria, gingivorrhagia, and hypogastric abdominal pain. At presentation, the physical examination revealed a stable patient (BP 127/76 mmHg, tachycardia (105 bpm), SaO_2_ 97% in A-a, mild tachypnea (23 breath/min)) afebrile, with purpuric lesions on the lower limbs, ecchymosis, and sensibility at the palpation of the abdomen in the hypogastric region. There were no palpable lymphadenopathies or visceromegalies. Laboratory examination at admission showed (Table 1) bicytopenia (severe normochromic, normocyte anemia, severe thrombocytopenia), mild leukocytosis with the predominance of neutrophils, lymphopenia, renal failure (creatinine clearance 30 mL/min/1.73 mp), mild hypopotassemia, mild inflammatory syndrome, elevated D-dimers, and low fibrinogen. Urine cultures were positive for Escherichia coli. Blood cultures were negative. Procalcitonin (PCT) was not elevated excluding sepsis (PCT 0.5 ng/mL means that sepsis is ruled out). The RT-PCR test for SARS-CoV-2 infection was positive.

**Figure 2:**
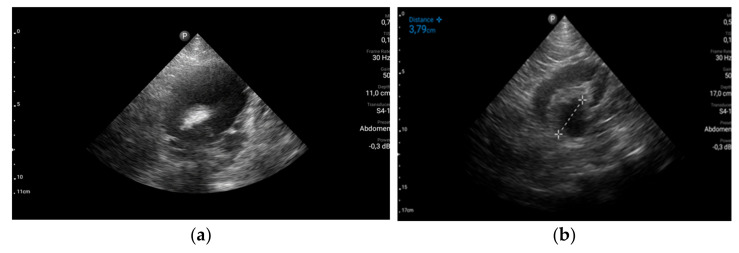
Abdominal ultrasound. (**a**) Hypogastric transversal section: urinary bladder obstructive stone, 3 cm in diameter and distended urinary bladder; (**b**) Right flank longitudinal section: third degree hydronephrosis of the right kidney.

The stage diagnosis was hemorrhagic syndrome precipitated by severe thrombocytopenia. To identify the causes and the consequences of the hemorrhagic syndrome, more laboratory tests and imagistic explorations were performed. Abdominal ultrasound revealed uncomplicated biliary stones and multiple urinary bladder obstructive stones with third degree bilateral hydronephrosis explaining the abdominal pain and renal failure (Figure 2). No visceral hematomas or ascites were detected.

**Figure 3:**
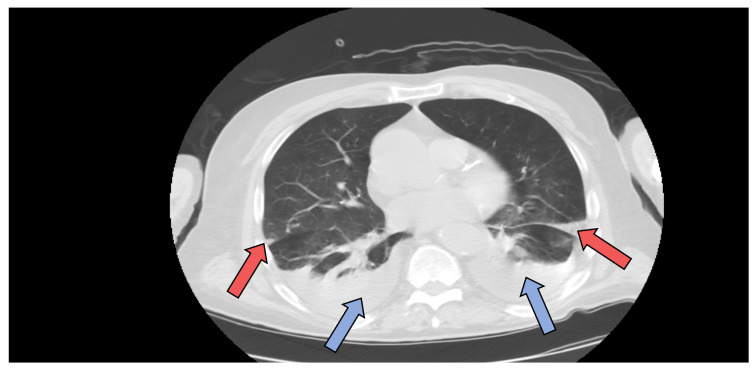
Chest computer tomography (CT) axial view showing bilateral pulmonary infarction (red arrows) and bilateral pleural effusion (blue arrows).

Due to the presence of COVID-19 infection, a native chest computer tomography (CT) was performed, which revealed bilateral pulmonary infarction (Figure 3 red arrows) and bilateral pleural effusion (Figure 3 blue arrows), but no signs of ground glass opacities characteristic of COVID-19 pneumonia. Lower limb deep venous ultrasound excluded deep vein thrombosis. The echocardiography did not show signs of high pressure in pulmonary arteries and troponins and NT-proBNP were in normal range, thus excluding a massive TEP.

A hematological examination was performed, which helped establish the diagnosis of thrombocytopenic purpura in the context of DIC and SARS-CoV-2 infection. Peripheral bone marrow puncture and lymphocyte immunophenotyping excluded a myeloproliferative or a lymphoproliferative disorder. The International Society of Thrombosis and Hemostasis (ISTH) criteria for the diagnosis of DIC were applied, obtaining a total score of 3 points, which was compatible with DIC [7]. Considering that the patient did not have a pre-existing liver disease and no significant liver pathological changes were described, and that no signs or symptoms of other organ or system damage were found (e.g., central nervous system, joint, renal), the DIC is most probably a consequence of COVID-19.

The final diagnosis was COVID-19 (mild respiratory form) complicated with pulmonary embolism; DIC with thrombocytopenic purpura; hemorrhagic syndrome; and severe anemia associated with obstructive renal failure, urinary infection with E. coli, and urinary bladder stones.

The management plan had multiple goals: correcting platelet count and anemia, treating obstructive renal failure and urinary infection, and preventing repetitive pulmonary embolism.

The treatment of DIC consisted of high-dose corticosteroid therapy (Dexamethasone 32 mg iv/day). For the correction of anemia, thrombocytopenia, and hemorrhagic syndrome, blood transfusion was administered (5 U erythrocyte mass isogroup, isoRh, 7 U fresh frozen plasma, 5 U platelets mass). In combination, proton pump inhibitors have been administered to prevent gastrointestinal bleeding in a patient with severe anemia and high doses of corticosteroids. A urinary catheter was inserted to relieve abdominal pain and correct obstructive renal failure. Even though the patient was diagnosed with bilateral pulmonary infarction, hemorrhagic syndrome, severe anemia, and severe thrombocytopenia are major contraindications for anticoagulant treatment, which is why no anticoagulant treatment was administered at the time of admission. For the bacterial infection, large spectra antibiotics were administered intravenously for 10 days in combination with probiotics.

During the hospitalization, the patient showed favorable development with the correction of the anemic syndrome and of the thrombocytopenia, the amelioration of the inflammatory syndrome, and the correction of the renal failure. Due to the favorable clinical and paraclinical development, it was decided to gradually decrease the dose of dexamethasone, but with the decrease in the, the patient showed a proportional decrease in thrombocytes, which required maintaining a high dose of dexamethasone during hospitalization and corticosteroids at home (80 mg Medrol, equivalent to 16 mg dexamethasone with tapering of the dose over the next 6 weeks). After 6 weeks, the platelet count was increased to the lower limit of normal range and Medrol was discontinued. Oral anticoagulant was administered after purpuric lesions disappeared and platelet count exceeded 100,000/mm^3^ [8]. Urological evaluation was recommended at discharge.

The particularities of this case are:

- Severe immune thrombocytopenic purpura was diagnosed along with DIC and severe hemorrhagic syndrome in a mild form of SARS-CoV-2 infection;
- Favorable development despite the worse prognosis;
- Good response to high doses of corticosteroids and no significant adverse reaction.

## 3. Discussion

Patients with COVID-19 infection present hematological changes depending on the patient’s immune response and the severity of the infection [1]. We presented above two different manifestations of thrombotic disorders related to COVID-19: one severe form of immune thrombocytopenia in a young woman with no comorbidities and a severe form of thrombocytopenia along with CID and acute urinary obstructive disease. Interestingly, both patients presented no signs of COVID-19 pneumonia.

According to specialty literature, mild thrombocytopenia is present in 45–55% of COVID-19 positive patients [9,10]. In a meta-analysis, which processed the clinical and paraclinical data of 5636 COVID-19-positive patients, it was concluded that there is a direct relationship between the clinical form of SARS-CoV-2 infection and the onset of thrombocytopenia. Thrombocytopenia occurs predominantly in patients with a severe form of SARS-CoV-2 infection [11].

Thrombocytopenia has been reported more frequently in patients with moderate or severe forms of COVID-19 infection. It usually occurs more than 10 days after the onset of symptoms [12]. Thus, thrombocytopenia may be a potential biomarker of negative prognosis in patients with COVID-19 [11]. There are several pathophysiological mechanisms that may be involved in the development of thrombocytopenia in patients with COVID-19 infection. In those with moderate or severe forms, the SARS-CoV-2 virus causes hyperinflammation and hypercoagulability. As a result, thrombocytopenia may be caused by platelet consumption during hypercoagulability and hyperinflammation.

For consumption thrombocytopenia, the risk factors are advanced age, male gender, high APACHE II score, neutropenia, lymphopenia, elevated CRP, and a low PaO_2_/FiO_2_ ratio. Thrombocytopenia can also be caused by a decrease in thrombopoietin (a regulator of megakaryopoiesis and platelet production) following damage caused to hepatocytes in SARS-CoV-2 infection [11]. In bone marrow, the viral infection of megakaryocytes can cause apoptosis and can reduce platelet maturation [11]. However, in the specialty literature, several cases have been presented showing that COVID-19 infection is associated with the onset or recurrence of immune thrombocytopenia (ITP), which is characterized by isolated thrombocytopenia, without any tendency to thrombosis [13,14]. The first case illustrates an ITP in a patient with a mild COVID-19 infection, who responded well to corticoid therapy. This severe immune thrombocytopenia in COVID-19 infection is a different entity, as it is milder than consumption thrombocytopenia, with a good prognosis. It can be associated with any degree of severity of COVID-19 infection, but it has been more commonly reported as associated with moderate and severe forms of the disease [15]. ITP is an autoimmune disease—with seasonal fluctuations—characterized by persistent thrombocytopenia secondary to autoantibodies absorbed on the surface of platelets, which generates premature destruction of the platelets by the reticuloendothelial system (especially the spleen) [16]. The mechanism of ITP in COVID-19 may be explained by molecular mimicry. The infection with the SARS-CoV-2 virus can cause antibodies to cross-act with certain platelet glycoproteins. Platelets coated with these antiplatelet antibodies will thus be eliminated by the reticuloendothelial system. These antibodies can also inhibit the development of bone marrow megakaryocytes and they can promote their apoptosis, thereby inhibiting platelet production [17].

Platelets do not have ACE2 receptors. However, a recent study suggests that platelets may take up SARS-CoV-2 mRNA independently/irrespective of ACE2. The diversification of an antigen-induced immune response to new T-cells and/or antibody specificities targeting new target epitopes of the same or different antigen is known as “epitope spread” [17]. CD8-positive cytotoxic T-cells can directly cause platelet lysis, induce platelet apoptosis, and inhibit platelet production by the maturation of megakaryocytes [18]. Low level or dysfunctional regulatory CD4-positive T-cells can also be seen in patients with ITP, indicating their possible role [19]. SARS-CoV-2 infection can lead to a recurrence of thrombocytopenia in patients with a history of thrombocytopenia caused by other diseases, such as systemic lupus erythematosus [20].

In adult patients, ITP has a sudden onset with variable symptoms ranging from nasal bleeding, gingivorrhagia (as in our cases), or purpuric skin lesions. Hemorrhages occur in rare cases of severe ITP when platelets number drops below 30,000/mmc; the lower the platelets, the higher the risk of major bleeding events such as cerebral bleeding (usually taking place if platelets are below 5000/mmc) [16]. There is no specific test for ITP; the presence of antiplatelet antibodies is not mandatory for a positive diagnosis. The clinical context, the presence of thrombocytopenia, the exclusion of other pathologies, and the response to corticoid therapy are usually enough for the diagnosis of ITP [16]. A short mention regarding the antiplatelet antibodies is in order. Antiplatelet antibodies target various platelet glycoproteins (GP), notably GPIIb/IIIa (fibrinogen receptor), GPIb/IX (von Willebrand factor), and, less frequently, GPIa/IIa (collagen receptor) and GPIV. Direct monoclonal antibody immobilization of platelet antigens assay (MAIPA) allows the detection of antiplatelet antibodies in up to 60% of ITP patients [21]. Unfortunately, antiplatelet antibody testing has low sensitivity and does not correlate with clinical outcomes. Some of the titers of antiplatelets antibodies are too low to be detected (false negative results). On the other hand, a positive result cannot be used as a unique diagnosis tool, but more as a screening test. Thus, these tests are not recommended to aid in the diagnosis or management of ITP [16]. It is worth mentioning that antiplatelet antibodies can be used to distinguish between immune and non-immune thrombocytopenia in complex cases associated with inappropriate platelet production in bone marrow and ITP, in cases of treatment-resistant ITP, or in cases of drug induced ITP [16].

Thus, ITP is a diagnosis of exclusion, which means that it is mandatory to exclude other viral infections that may cause thrombocytopenia. Exclusion should also be applied to other immune conditions that may cause thrombocytopenia. ITP is thought to be a consequence of viral infections of hepatitis B/C virus, cytomegalovirus, varicella zoster virus, HIV, and, more recently, secondary zika virus [22,23].

The appearance of ITP in the context of COVID-19 infection requires a positive diagnosis of SARS-CoV-2 infection. In our first case, the diagnosis of SARS-CoV-2 infection was made in concordance with international and national guidelines: in the epidemiological context, first of all, the detection of viral antigens through rapid diagnostic tests or Ag-RDTs is based on immunodiagnostic techniques and confirmed by the detection of viral RNA through automated nucleic acid amplification tests (NAAT) and reverse-transcription polymerase chain reaction (RT-PCR) [24]. No other laboratory testing is specific for COVID-19 positive diagnosis, but there are inflammatory markers that can be used for the classification of disease severity, such as D-dimer > 1000 ng/mL (normal range: <500 ng/mL), CRP > 100 mg/L (normal range: <8.0 mg/L), LDH > 245 units/L (normal range: 110 to 210 units/L), Troponin > 2 × the upper limit of normal (normal range for troponin T high sensitivity: females 0 to 9 ng/L; males 0 to 14 ng/L), Ferritin > 500 mcg/L (normal range: females 10 to 200 mcg/L; males 30 to 300 mcg/L), CPK > 2 × the upper limit of normal (normal range: 40 to 150 units/L), and lymphopenia [25]. These laboratory features are associated with severe disease and poor prognosis, and they have not been identified in our first presented case.

Most patients with ITP responded to the treatment with intravenous immunoglobulins and corticosteroids. Erythrocyte mass transfusions were performed in those with significant bleeding [19]. Mention must be made that the reported bleeding was rare, most of it being minor (epistaxis), while the intracranial bleeding was reported as being major.

It has been observed that elderly patients with multiple comorbidities are more prone to severe forms of COVID-19 and have a higher risk of thrombosis. Elevated levels of D-Dimers, although nonspecific, demonstrate an increased risk of thrombosis in patients with COVID-19, while decreased platelets, fibrinogen, or antithrombin are more commonly associated with an increased risk of DIC. In contrast, bleeding events have been reported less frequently in patients with COVID-19 [26]. The presence of thrombocytopenia at the onset of the disease is rare, and the number of platelets has been significantly lower in patients with severe disease [7]. It was also observed that the majority of patients who met the diagnostic criteria for DIC established by the ISTH did not survive (71.4%) and only 0.6% of patients with COVID-19 who met these criteria are among the survivors [7].

DIC is a diagnosis of exclusion, and the clinical picture and general condition of the patient were inconsistent with laboratory investigations, which is why, before establishing this diagnosis, all possible steps were taken to rule out other causes. In this sense, considering that the patient did not show central nervous system, joint, or kidney damage and was not known to have any underlying pathology, thrombocytopenic thrombosis, vasculitis, and autoimmune diseases were excluded. Imaging examinations and blood smear contributed to the exclusion of liver pathology, immune thrombocytopenia, and myelodysplastic syndromes, thus preserving a possible diagnosis of DIC in the context of SARS-CoV-2 infection. It appears that patients with COVID-19 rarely meet the diagnostic criteria for DIC. DIC is usually defined by the criteria of the ISTH [27]. The laboratory presentation of DIC from COVID-19 is different than that from bacterial sepsis or trauma. In COVID-19 the coagulation markers such as aPTT or PT prolongation are only mildly modified. Low platelet count, low fibrinogen levels, and markers of hyperfibrinolysis are not common [28]. This spectrum of coagulation changes is called COVID-19-associated coagulopathy and it has three stages [29]:

- Stage 1: elevated D-dimers;
- Stage 2: D-dimers and mild thrombocytopenia and/or mildly prolonged PT and aPTT;
- Stage 3: critical illness and classic DIC.

The prognosis of patients with COVID-19 appears to be closely related to platelet counts, as Asakura et al. showed in a study on 1476 patients, 16.1% of whom died. The authors found that the mortality rate was 92.1% for patients who had platelet counts of 0–50,000/mm^3^, 61.8% for those with counts of 50,000–100,000/mm^3^, 17.5% for those with counts of 100,000–150,000/mm^3^, and only 4.7% in patients with counts > 150,000/mm^3^ [30].

Another particularity of the second case is the absence of respiratory symptoms and pneumonia. In patient 2, the chest CT scan did not provide any sign of lung lesions manifested by ground glass opacities (GGO) typically found in COVID-19 pneumonia. As far as we know, chest CT scan has a higher sensitivity (98%) for COVID-19 pneumonia than even RT-PCR (70%) [31]. Therefore, some hospitals have used chest CT scans as a primary tool for the diagnosis of COVID-19. However, some authors found that about one quarter of the patients with clinical symptoms and positive RT-PCR findings have normal chest CTs, without typical GGO [32]. During these past two years of the pandemic, we have learned that COVID-19 is a polymorphic disease with manifestations ranging from typical GGO pneumonia to vascular, neurological, or digestive disease. In case 2, there were no respiratory symptoms identified during hospitalization which were consistent with the chest CT findings, explaining why no other investigation was performed. Bronchoscopy, is rarely used for the diagnosis of COVID-19; it is used only if nasopharyngeal swabs for RT-PCR are negative and there is another hypothesized infectious diagnosis that would significantly change the clinical management [33]. The indications for bronchoscopy in patients with SARS-CoV-2 infection are clearly stated and refer especially to intubated patients in the ICU: lung resistance increase, suspected alveolar hemorrhage, reposition of the tracheal tube, endoscopic tracheotomy assistance, difficult intubation assistance, suspected aspiration pneumonia, suspected tracheal injury, or suspected obstruction by secretions or clots [33].

## 4. Conclusions

The spectrum of hematological manifestation of COVID-19 is wide and frequently unpredictable. We presented two different extremes of severe thrombocytopenia (ITP and DIC), both precipitated by COVID-19 inflammation, without pulmonary manifestations and with good response to systemic corticosteroid therapy. Failure to diagnose it rapidly may lead to severe complications. Management with immunosuppressive corticosteroids in high doses should carefully balance the risk of bleeding versus deterioration due to infection.

## Figures and Tables

**Table 1:** Laboratory findings at admission.

Hematology	Coagulation	Inflammatory Syndrome
RBG 1.87 × 10^12^/L **Hb 5.6 g/d**L **Hct 16.8%** MCV 90.2 fl Reticulocytes 0.19 × 10^12^/L	aPTT 24.5 s INR = 1.28 PT = 14.3 s **D-dimers 1500 ng/mL** Fibrinogen 186 mg/dL	CRP = 1.06 mg/dL Ferritin = 445 ng/mL LDH = 306 U/L Procalcitonin 0.5 ng/ml
	Renal function	Blood chemistry
**Platelets 6000/mm^3^** **WBC 10,500/mm^3^**	**Crea =2.22 mg/d**L **Urea = 188 mg/d**L	ALT 33 U/L AST 28 U/L
**Neutrophiles 9860/mm^3^** **Lymphocytes 400/mm^3^**	**Cl crea = 30 mL/min/1.73 m^2^** **K = 3.27 mmol/**L	**Albumin 2.61 mg/d**L Bilirubin 0.5 mg/dL

ALT: alanine transaminase, APTT: activated partial thromboplastin time, AST: aspartate aminotransferase, Hb: Hemoglobin, Hct: hematocrit, INR: International normalized ratio, PT: Prothrombin time, K: potassium, RBG: red blood cells, WBC: white blood cells, CRP; C reactive protein, LDH: lactic dehydrogenase, MCV: medium cellular volume.

## Data Availability

Not applicable.
